# Clinical, Radiographic, and Molecular Analysis of Patients with X-Linked Hypophosphatemic Rickets: Looking for Phenotype–Genotype Correlation

**DOI:** 10.3390/diagnostics15010091

**Published:** 2025-01-03

**Authors:** Marco A. Olivas-Valdez, Armando Blanco-López, Daniela Velázquez-Arestegui, Teresita Vera-Zazueta, Douglas Colmenares-Bonilla, Lilian Reyes-Morales, Miguel A. Blanco-Uriarte, Lucero Monterde-Cruz, Alberto Hidalgo-Bravo

**Affiliations:** 1 Clínica Shriners Tijuana, Av. Paseo de Los Héroes 10999, Zona Río, Zona Urbana Rio Tijuana, Tijuana 22010, Mexico; marcoolivas@hotmail.com; 2 Hospital Shriners para Niños-México, Av. del Imán #257, Pedregal de Sta. Úrsula, Coyoacán, Ciudad de México 04600, Mexico; ablanco@shrinenet.org (A.B.-L.); dradanielava@me.com (D.V.-A.); miguelblanco92@gmail.com (M.A.B.-U.); 3 Hospital Ángeles Tijuana, Av. Paseo de Los Héroes 10999, Zona Río, Zona Urbana Rio Tijuana, Tijuana 22010, Mexico; tereverazazueta@gmail.com; 4 Servicio de Ortopedia Pediátrica, Hospital Regional de Alta Especialidad del Bajío, Blvd. Milenio 130, Col San Carlos la Roncha, León 37544, Mexico; douglas_cb@yahoo.com; 5 Departamento de Nefrología, Instituto Nacional de Pediatría, Av. Insurgentes Sur 3700 Letra C, Insurgentes Cuicuilco, Ciudad de México 04530, Mexico; dralilianreyes@gmail.com; 6 Star Medica-Hospital Infantil Privado, Nueva York 15, Col. Nápoles, Ciudad de México 03810, Mexico; 7 Departamento de Medicina Genómica, Instituto Nacional de Rehabilitación, Calzada México-Xochimilco 289, Col. Arenal de Guadalupe, Ciudad de México 14389, Mexico

**Keywords:** X-Linked hypophosphatemic rickets, *PHEX*, genotype–phenotype correlation, bone, XLH

## Abstract

Background/Objectives: X-linked hypophosphataemic rickets (XLH) represents the most frequent type of rickets from genetic origin, it is caused by mutations on the *PHEX* gene. The main clinical manifestations are short stature and bone deformities. Phenotype variation is observed at the intrafamily and interfamily level. The bases for this variation are not fully understood. The aim of this study was to investigate if there is a phenotype–genotype correlation in a cohort of patients with confirmed diagnosis of XLH. Methods: We recruited a total of 130 patients of Mexican Mestizo origin with confirmed molecular diagnosis of XLH; this is one of the largest cohorts reported. Results: Radiographies for calculating the rickets severity score (RSS) were available from 50 patients. A total of 56 different pathogenic variants were found among the study population; from them, 31 variants have not been previously reported. We compared the RSS values between individuals considering clinical and biochemical characteristics such as age, height, sex, phosphorus, and alkaline phosphatase in serum; no significant differences were observed. Then, we compared the RSS considering if the variant was intronic or exonic and considering the presence of a truncated protein or not. None of the two comparisons showed significant differences. Conclusions: We did not find a genotype–phenotype correlation in the study population. Despite the knowledge regarding the genetic cause of XLH, the mechanisms driving the intrafamily and interfamily variability remain elusive. More analyses looking for the genotype–phenotype correlation are necessary in other populations, especially considering the discovery of new mutations in patients from different origin.

## 1. Introduction

Rickets is a common disease mainly affecting the physes of long bones leading to a deficient mineralization of cartilage and osteoid. Therefore, there is an excessive accumulation of physeal cartilage, growth failure, and skeletal deformities [1,2]. Rickets can be classified based on the serum levels of phosphates and calcium, which also allows us to know the etiology [3]. The most frequent type of rickets from genetic origin is the X-linked hypophosphataemic rickets (XLH, MIM #307800) [3]. XLH has a worldwide estimated incidence of one per twenty thousand live births [4]; in approximately 80% of cases, there is one affected parent, while the remaining 20% occurs via de novo mutations [5,6]. XLH is caused by mutations on the *PHEX* gene located on the locus Xp22.11. The *PHEX* protein contains 749 amino acids; it is a transmembrane protein that belongs to the type II zinc-dependent endopeptidase family, which regulates the activation or degradation of peptide hormones [5]. *PHEX* is mainly expressed in osteoblasts, osteoclasts, and odontoblasts [6]. The interaction between *PHEX* and DMP1 (dentin matrix protein 1) and α5β3-integrin forms a trimeric complex on the osteocyte plasmatic membrane, which can regulate the expression of fibroblast growth factor type 23 (FGF23) [6]. *PHEX* mutations cause the loss of function of its product, leading to increased plasma levels of FGF23, which decreases the expression of sodium/phosphate IIa and IIc cotransporters (NaPi-II and NaPi-IIc) in the renal proximal tubule. The consequence of the lack of cotransporters is a reduced phosphate reabsorption causing hypophosphatemia, blocking renal vitamin D alfa-1-hydroxylase, and the inhibition of 24-hydroxylase [5,6,7]. XLH has complete penetrance and is characterized by intrafamilial and interfamilial clinical heterogeneity. The main clinical manifestations appear between six months to two years old, with genu varum deformity and delay of independent gait, secondary disproportionate short stature, bone and joint pain, joint stiffness, weakness, delay dentition, spontaneous tooth abscess, and enamel hypoplasia [5]. In the adulthood period, patients can develop periodontitis, abnormal loss of teeth at early age, pseudofractures, osteoarthritis, extraosseus calcifications, neurosensorial deafness, otosclerosis, and physical disability due to enthesopathy [8,9,10,11]. The diagnosis of XLH is based on clinical and radiographic features, and it is confirmed through the molecular analysis of the gene *PHEX*. The rickets severity score (RSS) is a method for assessing the severity of the disease through radiographic evaluation; RSS measures the degree of metaphyseal fraying, concavity, and the proportion of the growth plate affected in the wrists (radius and ulna) and knees (femur and tibia). The range of the RSS is −10. Zero points represents a lack of radiographic changes, while ten points shows extremely severity of the disease. RSS is also employed to evaluate treatment response in XLH patients [12,13,14,15].

To date, more than 720 mutations have been described in patients with XLH (http://www.hgmd.cf.ac.uk/ac/index.php, accessed on 9 October 2024). Interfamilial and intrafamilial clinical heterogeneity has been observed in individuals harboring the same genetic variant. Through the years, there have been retrospective studies looking for a phenotype–genotype correlation; however, a clear correlation has not been found [16,17,18]. One of the major challenges is how to categorize the different classes of pathogenic variants. A recurring strategy has been to classify pathogenic variants based on the region where the mutations occur, exon or intron, the consequence at the protein level, as truncating or no-truncating mutations, and the domain of the protein affected [17,19,20,21,22]. The aim of this study was to describe the clinical, biochemical, and radiological findings of a series of patients with confirmed diagnosis of XLH of Mexican Mestizo origin. In addition, we attempted to establish a genotype–phenotype correlation. To the best of our knowledge this is the first deport of a group of patients from Mexico, and this is one of the biggest series from a single population reported.

## 2. Materials and Methods

### 2.1. Study Population

We retrospectively included patients that are also pediatric orthopedic patients with confirmed clinical diagnosis of XLH from 2019 to 2022 from two different health institutions in Mexico: the Shriners Hospital for children, Mexico and Shriners Hospital for children, Tijuana. We included patients with molecular test of the *PHEX* gene reporting variants classified as pathogenic (PV), probably pathogenic (PPV), and variants of uncertain significance (VUS), according to the American College of Medical Genetics criteria. We reviewed the medical file from each patient to retrieve clinical, biochemical, and radiographic data obtained during the initial assessment, before the prescription of any modality of treatment for XLH. Nevertheless, not all patients had complete radiographs (wrists and knees) in the medical records. Patients lacking a knee radiograph at the first assessment, before treatment, were excluded from the genotype–phenotype correlation analysis. The study procedures comply with the Declaration of Helsinki (1964) and subsequent amendments. All procedures were approved by the institutional ethics committee from the Hospital Shriners para Niños-México, approval number CEI-2024-17.

### 2.2. Molecular Test

Saliva or buccal swab samples were sent to a private laboratory for molecular analysis, through next generation sequencing (NGS), using a panel of genes related to hypophosphatemia. The panel included the following 17 genes, *ALPL*, *CLCN5*, *CTND*, *CYP27B1*, *CYP2R1*, *DMP1*, *ENPP1*, *FAH*, *FAM20C*, *FGF23*, *FGFR1*, *GNAS*, *OCRL*, *PHEX*, *SLC34A1*, *SLC34A3*, and *VDR*. For those patients with a positive result in the gene *PHEX*, the molecular test was extended to the family members under suspicion of having undiagnosed XLH. Those patients’ relatives with a positive molecular test were also invited to participate in this study. All patients signed an informed consent form accepting the molecular test and a review of their medical file. Personal health information (PHI) was protected through anonymization and generation of a code for each patient by the principal investigator of each investigation site. Full details regarding the NGS procedures and analysis strategy can be found at the laboratory website (https://www.invitae.com/us/provider-faqs/tech-and-quality, accessed on 2 December 2024). Briefly, taken from the laboratory website, genomic DNA is enriched for targeted regions using a hybridization-based protocol and sequenced using Illumina technology. All targeted regions are sequenced with ≥50× depth. Reads are aligned to a reference sequence (GRCh37), and sequence changes are identified and interpreted in the context of a single clinically relevant transcript. The analysis includes coding sequence, 20 bp of flanking intronic sequence, and other specific genomic regions demonstrated to be causative of the disease at the time of assay design.

### 2.3. Rickets Severity Score

The RSS is a validated quantitative method for grading the affection of the wrists (radio and ulna) and knees (femur and tibia) in patients with rickets [14]. As described above, we included patients with knee radiographs, before receiving any kind of treatment, for RSS assessing. Briefly, the tibia and femur from each knee were evaluated separately, and the most affected tibia and femur were considered for the score. The scale in knees goes from 0 to 6, with 6 being the most severe affection. The score evaluates the degree of metaphyseal fraying and concavity and the proportion of the growth plate damaged. As previously described, an RSS < 1.5 or >1.5 was considered as mild or severe disease, respectively [14]. RSS was evaluated by two specialists in pediatric orthopedics, the evaluators were blinded for the clinical, biochemical, and genetic data of the patients included in this study.

### 2.4. Statistical Analysis

For descriptive statistics, quantitative variants are presented as means with standard deviation (SD) or medians with interquartile range. Data distribution was assessed using the Shapiro–Wilk normality test. For comparison of quantitative variants, a Student’s *t* test was used, when normal distribution was observed, or a Mann–Whitney U test was used when distribution was not normal. The qualitative variables were described as proportions or percentages. For proportion comparison chi-squared test was used. For association of co-variables of interest with dependent variables, a logistic regression model was performed, either adjusted or not adjusted for co-variables. Correlations between quantitative variables were investigated through Pearson correlation coefficient. The kappa statistic was used to evaluate variability between the two evaluators of RSS. All statistical analyses were performed using the Statistical Package for the Social Sciences (SPSS) V25 and GraphPad Prism V8 software. A *p* value < 0.05 was considered as statistically significant.

## 3. Results

We included a total of 130 patients with confirmed diagnosis of XLH through radiographic assessment and molecular analysis of the gene *PHEX*. In our cohort of patients, the age range, at molecular diagnosis, was from 9 months to 65 years old. The group of patients consisted of 54 adults, representing 41.5% (39 females and 15 males), and 76 children, representing 58.5% (52 girls and 24 boys). Considering the total number of patients, we observed a ratio of adult to children of 1:1.4 and a female to male ratio of 2.3:1. Index patients, i.e., patients who attended the clinic under suspicion of bone dysplasia, were 68 (52.3%), of the 130 patients included. From these 68 index cases, 32 (47%) corresponded to cases with known family history of XLH, and 27 (39%) were considered de novo cases because no clinical data of XLH were present in their parents. From nine patients (13%), no information regarding family history could be retrieved; therefore, we were unable to determine whether these are familial or de novo cases. From the 68 index cases, 50 patients, 18 boys (36%) and 32 girls (64%), had knees radiographies, for RSS assessment, before any kind of treatment for XLH was received.

Considering the 50 index cases in whom radiographies from the knee were available for RSS evaluation, the mean age at clinical diagnosis was 7.6 years old (range 2–22 years old). For this group of 50 index cases, the mean age of independent walking was 1.5 years old (range 1–2.3 years old). Lower limbs deformity was a common clinical finding, and almost all the patients had genu varum deformity. The mean height of the study population was 101.61 cm (range 75–155 cm), and almost all individuals were below the third percentile of height for age. When we investigated if there were family history of XLH, for each index cases, we found that sixteen (32%) were unique cases, and twenty-five (50%) had a family history with at least one affected relative. For nine cases (18%), it was not possible to obtain family information. Among the index patients with positive family history, we found a range from one to seven affected family members. In most of the affected relatives, XLH had not been previously suspected; they have been diagnosed only as hereditary rickets. We could only obtain radiographies from two of the affected relatives; the rest of them did not have radiographies for RSS assessment.

**Figure 1:**
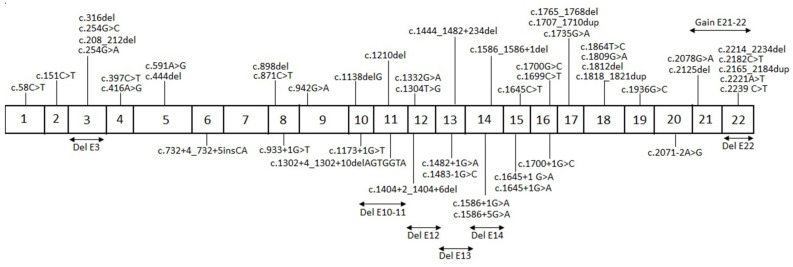
Schematic representation of the different pathogenic variants identified in *PHEX*. The above diagram depicts the variants lying on exons and resulting from duplications. The below diagram depicts the variants lying on intronic regions and resulting from deletions.

**Figure 2:**
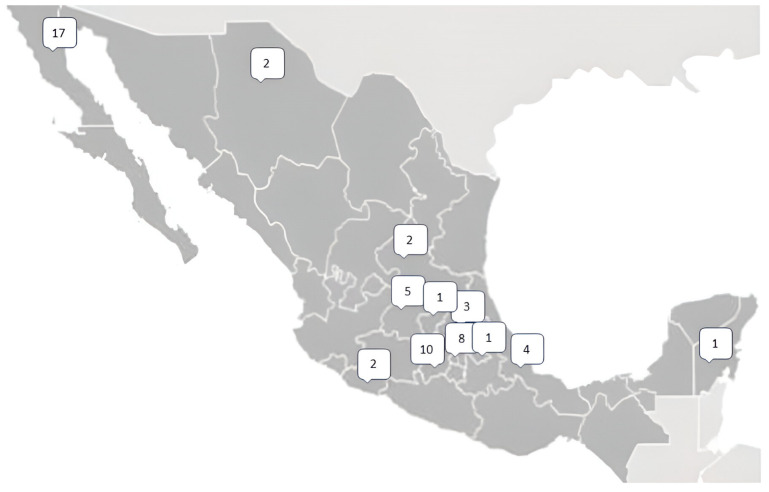
Map showing the distribution of the unique mutations over Mexico. Numbers represent the number of cases in each state. A plain map has been obtained from the publisher under the license commons: GNU Free Documentation License, version 1.2.

From the molecular results of *PHEX*, we found 56 different pathogenic variants (Supplementary Table S1). From the identified variants there were twenty frameshift variants, thirteen splicing variants, thirteen nonsense variants, nine missense variants, and one silent variant (Figure 1). Of these 56 unique pathogenic variants, 31 had not been previously reported; each of these variants was present solely in one family. The geographic distribution of gene variants along the Mexican territory is shown in Figure 2.

We attempted to establish a correlation between the phenotype and the clinical variables, as well as the genetic variants. For the correlation analysis, which included fifty index cases and two relatives, radiographies were obtained before the administration of any kind of treatment for XLH and identification of a pathogenic variant in the gene *PHEX*. Table 1 shows the demographic and molecular data of the 52 patients included in the correlation analysis.

**Figure 3:**
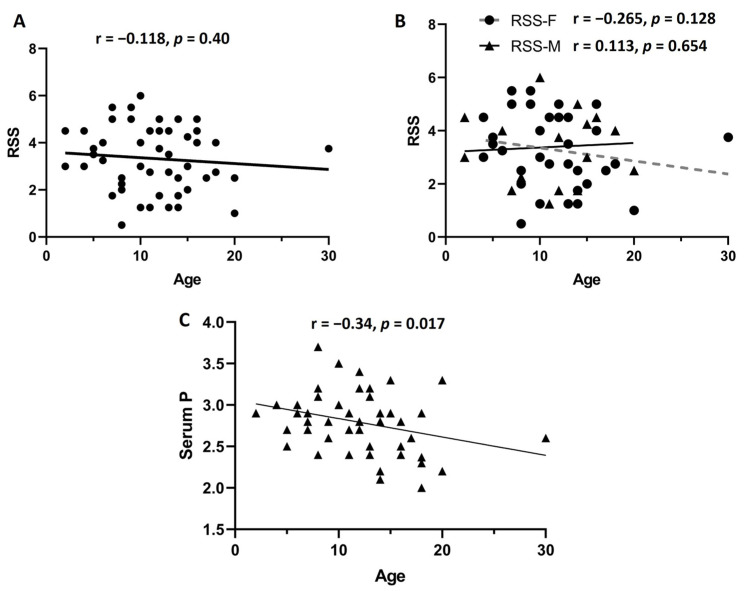
Correlation analysis. First, we analyzed the correlation between rickets severity score (RSS) in the 52 patients, and no significant correlation was observed (**A**). Afterwards, considering that males can have a more accelerated progression of the disease, we divided the group by sex (**B**). No significant correlation was observed in females (n = 34) or males (n = 18). When we performed correlation analysis between age and serum levels of phosphorus, a significant negative correlation was observed (**C**). Age is expressed in years.

The mean RSS value and age of these 52 patients included in the correlation analysis was 3.23 (1.34 SD) and 11.73 (5.23 SD), respectively. First, we considered the age of the patients, at the moment of radiographies obtention and suspicion of diagnosis, as a relevant factor potentially related to disease severity. We compared the RSS median values between individuals younger than 10 years old (n = 22, RSS median 11) versus individuals older than 10 years old (n = 30, RSS median 12). There was no statistically significant difference in RSS median values when comparing both age groups (*p* = 0.525). Additionally, we investigated if there was a correlation between the RSS and the age of the patients at the moment of the diagnosis of XLH. First, we analyzed the 52 patients; we did not observe a significant correlation between the RSS and age (r = −0.118, *p* = 0.40). Afterwards we performed the same correlation analysis between RSS and age, dividing the group of 52 patients by sex. However, this analysis did not reveal a significant correlation according to the sex of the patients (females n = 34, r = −0.265, *p* = 0.128; males n = 18, r = 0.113, *p* = 0.654) (Figure 3).

**Figure 4:**
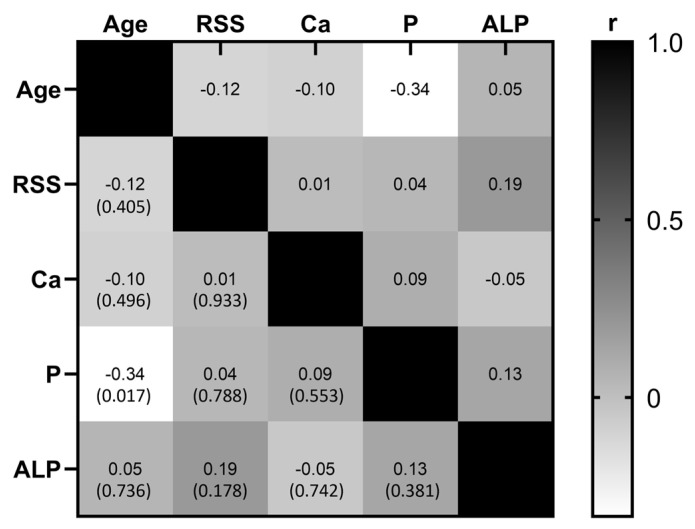
Correlation matrix. Correlation analysis was performed considering the age at diagnosis, rickets severity score (RSS), serum levels of calcium (Ca), phosphorus (P), and alkaline phosphatase (ALP). Grayscale represent the value of the correlation coefficient (r); positive correlations move towards the black color, while negative correlations move towards the white color, and the value of *p* is in parenthesis. The only significant correlation was observed between age and serum levels of phosphorus.

Furthermore, taking into consideration that males usually present more severe clinical manifestations, we further compared the RSS median values (3.5 for females and 3.37 for males) and height percentile between males (n = 18) and females (n = 34). Nevertheless, neither the RSS nor the height percentile showed statistically significant differences between the sexes (*p* = 0.754 and *p* = 0.998, respectively). In addition, we investigated if there was a correlation between the RSS and the levels of phosphorus, calcium, and alkaline phosphatase in serum. We performed a correlation analysis through a Pearson correlation coefficient; however, no significant correlation was observed between RSS and levels of phosphorus, calcium, and alkaline phosphatase (r = 0.039, *p* = 0.788; r = 0.012, *p* = 0.993; r = 0.194, *p* = 0.178, respectively). These results showed that none of the analyzed clinical or biochemical variables was affected or correlated with the RSS value in our study population. We attempted to investigate if there was a correlation between the biochemical and clinical features of this group of 52 patients. For this analysis we included age at diagnosis, levels of phosphorus, calcium, and alkaline phosphatase, all of them at the initial assessment before prescription of any kind of treatment. Figure 4 depicts the results of this correlation analysis; the only statistically significant correlation was observed between age at diagnosis and levels of phosphorus in serum (r = −0.335, *p* = 0.017); the remaining associations were not significant.

In our population study, we found 56 different variants in the *PHEX* gene causing XLH. It is important to point out that 31 out of these 56 variants, representing 55%, have not been previously reported. As mentioned above, fifty index cases and two relatives were included in the genotype–phenotype analysis; with this subgroup of patients, there were 26 previously unreported variants in the gene *PHEX*. For the genotype–phenotype analysis, different approaches were used. Previous studies have considered the localization of the genetic variants, whether they lie in an exon or an intron, for assessing the resulting phenotype. From our study population with an available RSS, we observed 40 mutational events affecting exons; these events included point mutations, small insertions, small deletions, or whole exon deletions. The remaining 12 individuals with an available RSS had a mutational event involving an intron; most of them were point mutations and some were small insertion/deletions. We compared the height percentile between variants affecting exons versus variants lying on introns; however, no statistically significant difference was observed (*p* = 0.537). We also compared the median values of the RSS between these two groups; however, no statistically significant difference was observed (*p* = 0.556). A second approach considered the outcome of the mutational event at the protein level; it describes whether the mutations led to a truncated protein or a non-truncated protein. Of the 52 patients included in the correlation analysis, 13 harbor missense point mutations, leading to non-truncated proteins. The remaining 39 patients harbor either nonsense mutations, splicing mutations, or insertions/deletions, all of them leading to truncated proteins. We compared the height percentile between patients with variants leading to a truncated protein versus those with variants leading to a non-truncated protein; this comparison did not reveal a statistically significant difference (*p* = 0.107). Additionally, we compared the RSS between these two mutation categories; however, no statistically significant difference was observed (*p* = 0.189). Afterwards, we used a regression model for investigating if the location of the mutational event, exonic or intronic, or the outcome at the protein level, truncated protein versus non-truncated protein, had an effect on the RSS value. The localization of the genetic variant, exonic or intronic, did not show association with the RSS value (OR 0.090, CI −0.612–1.183, *p* = 0.526). Regarding the outcome at the protein level, the analysis showed no significant association with the RSS value (OR 1.62, CI 0.166–15.95, *p* = 0.677). Our analysis did not find a genotype–phenotype correlation considering the localization of the pathogenic variant or the consequence at the protein level. A third strategy previously used in other studies considers two regions of the protein, the first spanning from the 5′ end of *PHEX* up to amino acid residue 649 in exon 19 and the second beyond amino acid 649. The rationale behind this division is because the highly conserved transmembrane domain and the two zinc-binding motifs are comprised in the region comprised between the 5′ end and amino acid residue 649. Furthermore, this region also contains seven highly conserved cysteine residues [17]. From the individuals harboring missense mutations, i.e., mutations leading to non-truncated proteins, only one was allocated beyond amino acid residue 649; therefore, this last strategy was not applied to our study population.

## 4. Discussion

X-Linked hypophosphatemic rickets (XLH) is the most common form of genetic rickets, with an estimated incidence of one in twenty thousand live births worldwide [4]. XLH is caused by mutations in the *PHEX* gene located on the X chromosome. Despite being an X-linked disorder, it is possible to find males and females that are severely affected by it. Herein we present the largest series of Mexican Mestizo patients with confirmed molecular diagnosis of XLH.

According to the literature, up to 80% of XLH cases have a positive family history of the disease [23,24]. In our study population, we observed positive family history only in 50% of index cases, while the percentage of de novo cases was 32%. In 18% of the cases, no family history could be retrieved. This different behavior could be related to the lack of information about the disease among health professionals. The average time for diagnosis observed in the index cases exceeds seven years. In some families, the diagnosis of the affected parent and other relatives was established after the diagnosis of the index case. This is not an uncommon situation; in other cohorts of patients, the diagnosis of parents or other relatives has also been confirmed after diagnosis of an index case [18]. Therefore, it is possible that the missing information regarding the parents or other family members simulates the absence of affected relatives. In addition, the lack of recognition of the disease in the relatives is an important cause of diagnosis delay and, therefore, adequate treatment. It is important to identify the relatives at risk through a careful medical interrogation, while always bearing in mind that an early treatment can prevent complications associated with XLH. Taken together, these data points identify at least two factors related to the underdiagnosis of XLH, the delay of an accurate diagnosis and the lack of awareness of this condition among health professionals. The ultimate result of this situation is the appearance of disease-related complications which impact the quality of life of the patients and their families.

The gold standard for clinical diagnosis of XLH are the radiographs of the knee and wrist; they also allow us to assess the severity of the disease. Radiographic changes comprised abnormal bone mineralization and growth plate ossification [14]. These changes occur mainly at the metaphysis of distal radius and ulna, distal femur, and proximal tibia. RSS is a quantitative method for assessing the affection of knees and wrists in XLH patients [17]. We investigated whether clinical or biochemical variables could be associated with RSS values. We compared the median values of RSS between individuals younger than 10 years old versus those older than 10 years old; nevertheless, no significant differences were observed. Afterwards, we compared the RSS between male and female patients, but no significant difference was observed. This finding agrees with previously published studies [18]. Nevertheless, in a Norwegian cohort of patients, a more severe skeletal disease was observed that affected males compared to female patients [20]. For a better understanding of the severity of the disease between sexes, it is necessary to enrich the current clinical evidence. Furthermore, the correlation analysis between RSS and alkaline phosphatase and serum levels of phosphate did not reveal significant results. Serum levels of phosphate have been previously correlated with the height and the sitting height index. This evidence points out the relevance of phosphate serum as an indicator of growth when treatment is established [25]. We explored the possibility of correlation between other biochemical and clinical variables related to XLH. We observed a significant correlation between age and levels of phosphorus in serum. Although it is a weak correlation, this finding is in agreement with the characteristic of phosphate lost through the kidney in XLH patients, which seems to be more severe in older patients with no treatment.

Different research groups have looked for a genotype–phenotype correlation among XLH patients; however, results are not consistent. We considered different strategies for investigating whether there was a genotype–phenotype correlation. In the first strategy, we divided the patients into two groups depending on whether the variant was located within an exon or within an intron. The second strategy is widely used; the patients are divided into two groups depending on the consequence of the variant at the protein level, one group where the result is a truncated protein and another where the result is a non-truncated protein. Both strategies were used to look for a correlation with height or RSS value; nevertheless, no significant results were found. In a previous study, Zheng et al. concluded that severity of XLH does not corelate with gene variants leading to truncated proteins or non-truncated proteins [26]. Although, there are other studies suggesting the existence of a phenotype–genotype correlation between some variables. Jimenez et al. reported a statistically significant association between lower height Z-score and the presence of variants causing truncated proteins (Z score—2.7 vs. −1.3, *p* < 0.05) [19]. Additionally, Zhang et al. reported that patients who have variants in the N-terminal domain of the *PHEX* protein have earlier age of onset of XLH (*p* = 0.015) and a higher concentration of intact FGF23 compared to individuals with variants at the C-terminal domain [27]. In our study population, among individuals harboring missense mutations, only one patient has a mutation within the C-terminal domain. Therefore, this last approach could not be conducted in our analysis. It has been difficult to describe a phenotype–genotype correlation, partially derived from the limited number of patients included in many studies in different populations [28]. There is a study where the authors performed an initial analysis during the first medical interview, and they performed a second analysis after a follow-up period. They did not find a significant correlation at the initial assessment, but they observed a significant correlation with serum phosphate, nephrocalcinosis, and the number of orthopedic surgeries after the follow-up period. Additional studies with larger cohorts and a prospective follow-up are needed to unravel the existence of a genotype–phenotype correlation. Another factor which complicates the determination of a phenotype–genotype correlation is the large number and variation in pathogenic variants reported along the *PHEX* gene, and that some of them have been reported only in one individual or one family. Herein we found a total of 56 different mutations in the *PHEX* gene causing XLH; within the subgroup of patients included in the genotype–phenotype analysis, we observed 26 variants not previously reported. This large variation in gene variants and the fact that some of them have been reported in only one family makes it difficult to categorize the large variety of mutational events in a single cohort.

Our study has some limitations; wrist and knee radiographies were not obtained for all patients at the time of diagnosis before the initiation of any kind of therapy for XLH. In addition, only knees radiographies were evaluated in a subgroup of our study population. Nevertheless, the knee radiographies were evaluated by experts with an extensive experience treating XLH patients, and we gather an appropriate number of subjects for this analysis. Relatives of index cases did not have biochemical or radiographic data. As mentioned before, in some families the affected progenitor of the index case, or other relatives, were diagnosed after the molecular test of the index case was obtained. One strength of our study is the large number of individuals with confirmed diagnosed of XLH; this number of patients bring confidence to our results. Another strength is that none of the patients included in the correlation analysis had received treatment before the obtention of the radiographies. An additional strength is the number of variants not previously reported. This information enriches the allele pool causing XLH; this knowledge is useful to delineate the presence of mutation hot spot in *PHEX*. The accumulation of more information regarding the mutational variation in the gene *PHEX* increases the possibility to identify a genotype–phenotype correlation.

## 5. Conclusions

In conclusion, it is important to spread the awareness regarding XLH among the health professionals and to have access to molecular test in order to reduce the delay of diagnosis of the index cases and their relatives. An effective measure for early and accurate diagnosis may represent a reduction in the human and economic burden caused by XLH. On the other hand, so far there is no clear genotype–phenotype correlation; however, larger cohorts and clinical follow-up can be effective strategies for a better unraveling of a possible genotype–phenotype correlation. Our data provide clinical and molecular data for enriching the body of evidence for a better comprehension of the clinical behavior of XLH. The contribution of new reports will unravel whether a genotype–phenotype correlation can be established. The ultimate aim is to improve the medical management of patients affected by XLH.

## Figures and Tables

**Table 1:** Clinical data of the study population included in the correlation analysis.

ID	Sex	Variant	Consequence	Age	RSS	Ca mg/dL	P mmol/L	ALP U/L
Px 4	F	c.2239 C > T (p.Arg747*)	Nonsense	10	1.25	139.4	3	455.6
Px 5	M	c.1700 + 1G > C (p.?)	Splice variant	2	3	140.8	2.9	379.6
Px 7	M	c.1302 + 4_1302 + 10delAGTGGTA (p.?)	Splice variant	2	4.5	139.6	2.9	394.4
Px 14	M	c.1483-1G > C (p.?)	Splice variant	16	4.5	139.6	2.4	252.7
Px 35	F	c.1735G > A (p.Gly579Arg)	Missense	8	0.5	136.2	2.4	800
Px 37	F	c.1645 + 1 G>A	Splice variant	30	3.75	138.8	2.6	647.5
Px 44	F	c.1735G > A (p.Gly579Arg)	Missense	17	2.5	10	2.6	623
Px 45	F	c.1707_1710dup (p.Tyr571Glufs*12)	Frameshift	9	5.5	9.5	2.8	420
Px 46	F	c.1304T > G (p.Met435Arg)	Missense	8	2.5	10.8	3.1	487
Px 48	M	Deletion exon 22	Frameshift	11	1.25	9.3	2.9	427
Px 50	F	c.1735G > A (p.Gly579Arg)	Missense	13	2.75	9.3	2.5	539
Px 51	F	c.1586 + 5G>A	Splice variant	16	4	10.1	2.8	581
Px 52	F	c.444del (p.Ile148Metfs*73)	Frameshift	13	1.25	9.9	3.1	459
Px 53	M	c.871C > T (p.Arg291*)	Nonsense	15	4.25	9.3	3.3	606
Px 57	M	c.1765_1768del (p.Asn589Valfs*29)	Frameshift	6	4	9.7	2.9	557
Px 59	F	c.2221A > T (p.Arg741*)	Nonsense	15	2	10	2.9	512
Px 60	M	c.58C > T (p.Arg20*)	Nonsense	15	3	9.6	2.9	571
Px 61	F	Deletion (Exon 3)	Frameshift	14	1.25	9.2	2.8	404
Px 62	F	c.1645C > T (p.Arg549*)	Nonsense	10	4	NA	NA	NA
Px 65	F	c.1936G > C (p.Asp646His)	Missense	6	3.25	10	3	837
Px 66	M	c.942G > A (p.Trp314*)	Missense	7	1.75	9.6	2.9	355
Px 69	M	c.2125del (p.Ala709Leufs*31)	Frameshift	20	2.5	9.9	3.3	418
Px 71	M	c.1332G > A (p.Trp444*)	Nonsense	8	2.25	9.3	3.2	525
Px 73	F	c.1332G > A (p.Trp444*)	Nonsense	4	3	NA	NA	NA
Px 74	F	c.1645 + 1G>A	Splice variant	18	2.75	7.4	2	1155
Px 76	F	c.2165_2184dup (p.Lys729Valfs*18)	Frameshift	16	5	8.5	2.5	1363
Px 78	M	c.2182C > T (p.Gln728*)	Nonsense	10	6	10	3.5	754
Px 80	F	c.1735G > A (p.Gly579Arg)	Missense	10	4	9.9	3	568
Px 81	M	c.1404 + 2_1404+6del	Splice variant	18	4	9.5	2.9	464
Px 82	M	c.208_212del (p.Val70Serfs*7)	Frameshift	18	4	9.74	2.37	525
Px 84	F	c.416A > G (p.Tyr139Cys)	Missense	8	2	9.9	3.7	728
Px 91	F	c.254G > C (p.Cys85Ser)	Missense	11	4.5	10	2.7	442
Px 92	F	Deletion exon 14	Frameshift	7	5	9.1	2.8	578
Px 93	F	c.316del (p.Trp106Glyfs*2)	Frameshift	9	5	9.7	2.6	417
Px 94	F	c.1699C > T (p.Arg567*)	Nonsense	5	3.5	10.1	2.5	587
Px 95	F	c.1586_1586 + 1del (Splice site)	Splice variant	12	5	10.1	3.2	854
Px 96	M	c.1735G > A (p.Gly579Arg)	Missense	12	1.75	9	2.7	367
Px 97	F	c.1586+1G > A (Splice donor)	Splice variant	13	3.5	9.9	2.4	389
Px 98	M	Deletion (Exon 12)	Frameshift	14	1.75	9.7	2.1	1042
Px 101	F	c.1735G > A (p.Gly579Arg)	Missense	14	2.5	9.5	2.9	527
Px 102	F	c.1173+1G > T (Splice donor)	Splice variant	11	2.75	9.7	2.4	519
Px 103	M	c.933+1G > T (Splice donor)	Splice variant	14	5	9.5	2.2	530
Px 104	F	c.1586+5G > A (Intronic)	Splice variant	10	3	9.5	3.5	453
Px 105	M	c.2214_2234del (p.Met739_Ser745del)	Frameshift	12	3.75	9.6	3.4	599
Px 106	M	c.2078G > A (p.Cys693Tyr)	Missense	18	4	10.1	2.3	325
Px 107	F	c.1864T > C (p.Tyr622His)	Missense	4	4.5	9.4	3	454
Px 109	F	c.1700G > C (p.Arg567Pro)	Missense	7	5.5	9.8	2.7	507
Px 112	F	Deletion (Exons 10-11)	Frameshift	5	3.75	9.2	2.7	485
Px 116	F	c.1444_1482+234del	Frameshift	12	4.5	9.3	2.8	777
Px 117	F	c.1444_1482+234del	Frameshift	13	4.5	9.5	3.2	726
Px 128	F	c.591A > G (Silent)	Silent	20	1	9.6	2.2	293
Px 129	F	c.732+4_732 + 5insCA	Splice variant	14	1.75	9.3	2.2	205

ID, patient identification. RSS, rickets severity score. F, female. M, male. * Age in years at diagnosis. P, levels of phosphorus in serum. Ca, levels of calcium in serum. ALP, alkaline phosphatase. In italics, members of the same family.

## Data Availability

Data are available upon reasonable request to the corresponding authors.

## Supplementary Materials

The following supporting information can be downloaded at the following website: https://www.mdpi.com/article/10.3390/diagnostics15010091/s1, Table S1: Description of gene variants found in the study population.
