# Supplementary material for: Clinical, Radiographic, and Molecular Analysis of Patients with X-Linked Hypophosphatemic Rickets: Looking for Phenotype–Genotype Correlation
Source: Diagnostics (Basel). 2025 Jan 3;15(1):91. doi: 10.3390/diagnostics15010091 (PMC11719845; doi:10.3390/diagnostics15010091)
Supplement: Supplementary file 1 [file diagnostics-15-00091-s001.zip › diagnostics-3288237-supplementary.pdf]

Suppl. Table 1. Description of gene variants found in the study population.

Patient ID	Mexico's state	Index case/Relative	Mutation	Previously reported*
Px 1	Baja California	Index	c.1482+1G>A	No
Px 2	Baja California	Index	c.151C>T (p.Gln51*)	Yes
Px 3	Baja California	Index	c.1818_1821dup (p.Glu608Ilefs*6)	No
Px 4	Baja California	Index	c.2239 C>T (p.Arg747*)	Yes
Px 5	Baja California	Index	c.1700+1G>C (p.?)	No
Px 6	Baja California	Relative	c.1700+1G>C (p.?)	No
Px 7	Baja California	Index	c.1302+4_1302+10del	No
Px 8	Baja California	Relative	c.1302+4_1302+10del	No
Px 9	Baja California	Relative	c.1302+4_1302+10del	No
Px 10	Baja California	Relative	c.1302+4_1302+10del	No
Px 11	Baja California	Relative	c.1302+4_1302+10del	No
Px 12	Baja California	Relative	c.1302+4_1302+10del	No
Px 13	Baja California	Relative	c.1302+4_1302+10del	No
Px 14	Baja California	Index	c.1483-1G>C (p.?)	No
Px 15	Baja California	Relative	c.1483-1G>C (p.?)	No
Px 16	Baja California	Relative	c.1483-1G>C (p.?)	No
Px 17	Baja California	Relative	c.1483-1G>C (p.?)	No
Px 18	Baja California	Relative	c.1483-1G>C (p.?)	No
Px 19	Baja California	Relative	c.1483-1G>C (p.?)	No
Px 20	Baja California	Relative	c.1483-1G>C (p.?)	No
Px 21	Baja California	Relative	c.1483-1G>C (p.?)	No
Px 22	Baja California	Index	c.254G>A (p.Cys85Tyr)	Yes
Px 23	Baja California	Index	c.1699C>T (p.Arg567*)	Yes
Px 24	Baja California	Index	c.1812del (p.Thr605Leufs*14)	No
Px 25	Baja California	Relative	c.1812del (p.Thr605Leufs*14)	No
Px 26	Baja California	Relative	c.1812del (p.Thr605Leufs*14)	No
Px 27	Baja California	Relative	c.1812del (p.Thr605Leufs*14)	No
Px 28	Sinaloa	Relative	c.1812del (p.Thr605Leufs*14)	No
Px 29	Sinaloa	Relative	c.1812del (p.Thr605Leufs*14)	No
Px 30	Sinaloa	Relative	c.1812del (p.Thr605Leufs*14)	No
Px 31	Sinaloa	Relative	c.1812del (p.Thr605Leufs*14)	No
Px 32	Baja California	Index	c.1586_1586+1del (p.?)	No
Px 33	Baja California	Index	Gain exon 21-22	No
Px 34	Baja California	Relative	Gain exon 21-22	No
Px 35	Baja California	Index	c.1735G>A (p.Gly579Arg)	Yes
Px 36	Baja California	Relative	c.1735G>A (p.Gly579Arg)	Yes
Px 37	Baja California	Index	c.1645+1 G>A	Yes
Px 38	Baja California	Relative	c.1645+1 G>A	Yes
Px 39	Baja California	Index	c.1138delG (p.Arg380Glyfs*13)	No
Px 40	Baja California	Relative	c.1138del (p.Arg380Glyfs*13)	No
Px 41	Baja California	Index	c.1700G>C (p.Arg567Pro)	Yes
Px 42	Baja California	Relative	c.1809G>A (p.Trp603*)	Yes
Px 43	Sonora	Relative	c.1809G>A (p.Trp603*)	Yes
Px 44	Unknown	Index	c.1735G>A (p.Gly579Arg)	Yes
Px 45	Veracruz	Index	c.1707_1710dup (p.Tyr571Glufs*12)	No
Px 46	Estado de Mexico	Index	c.1304T>G (p.Met435Arg)	No

Px 47	Estado de Mexico	Relative	c.1304T>G (p.Met435Arg)	No
Px 48	Hidalgo	Index	Deletion exon 22	No
Px 49	Hidalgo	Relative	Deletion exon 22	No
Px 50	Unknown	Index	c.1735G>A (p.Gly579Arg)	Yes
Px 51	Veracruz	Index	c.1586+5G>A	Yes
Px 52	Quintana Roo	Index	c.444del (p.Ile148Metfs*73)	No
Px 53	Hidalgo	Index	c.871C>T (p.Arg291*)	Yes
Px 54	Hidalgo	Relative	c.871C>T (p.Arg291*)	yes
Px 55	Hidalgo	Relative	c.871C>T (p.Arg291*)	yes
Px 56	Hidalgo	Relative	c.871C>T (p.Arg291*)	yes
Px 57	Estado de Mexico	Index	c.1765_1768del (p.Asn589Valfs*29)	No
Px 58	Estado de Mexico	Relative	c.1765_1768del (p.Asn589Valfs*29)	No
Px 59	Mexico City	Index	c.2221A>T (p.Arg741*)	No
Px 60	Michoacán	Index	c.58C>T (p.Arg20*)	Yes
Px 61	Estado de Mexico	Index	Deletion exon 3	Yes
Px 62	Mexico City	Index	c.1645C>T (p.Arg549*)	Yes
Px 63	Mexico City	Relative	c.1645C>T (p.Arg549*)	yes
Px 64	Mexico City	Relative	c.1645C>T (p.Arg549*)	yes
Px 65	Unknown	Index	c.1936G>C (p.Asp646His)	No
Px 66	Guanajuato	Index	c.942G>A (p.Trp314*)	Yes
Px 67	Guanajuato	Relative	c.942G>A (p.Trp314*)	yes
Px 68	Guanajuato	Relative	c.942G>A (p.Trp314*)	yes
Px 69	Mexico City	Index	c.2125del (p.Ala709Leufs*31)	No
Px 70	Mexico City	Relative	c.2125del (p.Ala709Leufs*31)	No
Px 71	Estado de Mexico	Index	c.1332G>A (p.Trp444*)	Yes
Px 72	Estado de Mexico	Index	c.1332G>A (p.Trp444*)	Yes
Px 73	Estado de Mexico	Relative	c.1332G>A (p.Trp444*)	Yes
Px 74	Mexico City	Index	c.1645+1G>A	Yes
Px 75	Mexico City	Relative	c.1645+1G>A	yes
Px 76	Mexico City	Index	c.2165_2184dup (p.Lys729Valfs*18)	No
Px 77	Mexico City	Relative	c.2165_2184dup (p.Lys729Valfs*18)	No
Px 78	Guanajuato	Index	c.2182C>T (p.Gln728*)	No
Px 79	Guanajuato	Relative	c.2182C>T (p.Gln728*)	No
Px 80	Tlaxcala	Index	c.1735G>A (p.Gly579Arg)	Yes
Px 81	Chihuahua	Index	c.1404+2_1404+6del	No
Px 82	Veracruz	Index	c.208_212del (p.Val70Serfs*7)	No
Px 83	Veracruz	Relative	c.208_212del (p.Val70Serfs*7)	No
Px 84	Mexico City	Index	c.416A>G (p.Tyr139Cys)	No
Px 85	Mexico City	Relative	c.416A>G (p.Tyr139Cys)	No
Px 86	Mexico City	Relative	c.416A>G (p.Tyr139Cys)	No
Px 87	Mexico City	Relative	c.416A>G (p.Tyr139Cys)	No
Px 88	Mexico City	Relative	c.416A>G (p.Tyr139Cys)	No
Px 89	Mexico City	Relative	c.416A>G (p.Tyr139Cys)	No
Px 90	Mexico City	Relative	c.416A>G (p.Tyr139Cys)	No
Px 91	Michoacán	Index	c.254G>C (p.Cys85Ser)	No
Px 92	San Luis Potosi	Index	Deletion exon 14	No
Px 93	Veracruz	Index	c.316del (p.Trp106Glyfs*2)	No
Px 94	Mexico City	Index	c.1699C>T (p.Arg567*)	Yes
Px 95	Unknown	Index	c.1586_1586+1del (Splice site)	No

Px 96	Michoacán	Index	c.1735G>A (p.Gly579Arg)	Yes
Px 97	Tlaxcala	Index	c.1586+1G>A (Splice donor)	Yes
Px 98	Estado de Mexico	Index	Deletion (Exon 12)	Yes
Px 99	Estado de Mexico	Relative	Deletion (Exon 12)	Yes
Px 100	Estado de Mexico	Relative	Deletion (Exon 12)	Yes
Px 101	Estado de Mexico	Index	c.1735G>A (p.Gly579Arg)	Yes
Px 102	Estado de Mexico	Index	c.1173+1G>T (Splice donor)	Yes
Px 103	Hidalgo	Index	c.933+1G>T (Splice donor)	Yes
Px 104	Estado de Mexico	Index	c.1586+5G>A (Intronic)	No
Px 105	Guerrero	Index	c.2214_2234del (p.Met739_Ser745del)	No
Px 106	San Luis Potosi	Index	c.2078G>A (p.Cys693Tyr)	Yes
Px 107	Chihuahua	Index	c.1864T>C (p.Tyr622His)	Yes
Px 108	Chihuahua	Relative	c.1864T>C (p.Tyr622His)	Yes
Px 109	Michoacán	Index	c.1700G>C (p.Arg567Pro)	Yes
Px 110	Michoacán	Relative	c.1700G>C (p.Arg567Pro)	Yes
Px 111	Michoacán	Relative	c.1700G>C (p.Arg567Pro)	Yes
Px 112	Estado de Mexico	Index	Deletion (Exons 10-11)	Yes
Px 113	Estado de Mexico	Relative	Deletion (Exons 10-11)	Yes
Px 114	Estado de Mexico	Relative	Deletion (Exons 10-11)	No
Px 115	Estado de Mexico	Index	Partial deletion exon 13	No
Px 116	Estado de Mexico	Index	c.1444_1482+234del	No
Px 117	Estado de Mexico	Relative	c.1444_1482+234del	No
Px 118	Guanajuato	Index	c.898del (p.Met300*)	No
Px 119	Guanajuato	Relative	c.898del (p.Met300*)	No
Px 120	Guanajuato	Relative	c.898del (p.Met300*)	No
Px 121	Guanajuato	Relative	c.898del (p.Met300*)	No
Px 122	Guanajuato	Index	c.1210del (p.Asp404Thrfs*4)	No
Px 123	Guanajuato	Index	c.397C>T (p.Gln133*)	Yes
Px 124	Queretaro	Index	c.2071-2A>G	Yes
Px 125	Queretaro	Relative	c.2071-2A>G	Yes
Px 126	Mexico City	Index	c.591A>G (Silent)	Yes
Px 127	Mexico City	Relative	c.591A>G (Silent)	Yes
Px 128	Mexico City	Index	c.591A>G (Silent)	Yes
Px 129	Mexico City	Index	c.732+4_732+5insCA	No
Px 130	Mexico City	Index	c.1645C>T (p.Arg549*)	Yes

*Previously reported gene variants were searched in gnomAD, The Human Gene Mutation Database and Leiden Open Variation Database (LOVD)
